# Cross-Cultural Adaptation, Validity and Reliability of the European Portuguese Version of the Kerlan-Jobe Orthopaedic Clinic Shoulder & Elbow Score (KJOC)

**DOI:** 10.3390/healthcare13233081

**Published:** 2025-11-26

**Authors:** Gonçalo Almeida, Luísa Amaral, Rui Vilarinho, Bárbara Magalhães, Fátima Silva, Verónica Abreu, André Magalhães, Mário Esteves, Mariana Cervaens

**Affiliations:** 1 FP-I3ID, FP-BHS, Escola Superior de Saúde Fernando Pessoa, 4200-256 Porto, Portugal; lamaral@ufp.edu.pt (L.A.); rvilarinho@ufp.edu.pt (R.V.); fsilva@ufp.edu.pt (F.S.); andrem@ufp.edu.pt (A.M.); estevesm@ufp.edu.pt (M.E.); cervaens@ufp.edu.pt (M.C.); 2 CIR, E2S, Polytechnic of Porto, 4200-072 Porto, Portugal; 3 Health Research Network (RISE-HEALTH), Center for Translational Health and Medical Biotechnology Research (TBIO), E2S, Polytechnic of Porto, 4200-072 Porto, Portugal; bnm@ess.ipp.pt; 4 Research Center in Physical Activity, Health and Leisure (CIAFEL), Faculty of Sports, University of Porto (FADEUP), 4099-002 Porto, Portugal; 5 INSIGHT Piaget Research Centre for Ecological Human Development, Instituto Piaget, 4405-678 Vila Nova de Gaia, Portugal; 6 RISE-HEALTH, Fernando Pessoa University, 4249-004 Porto, Portugal

**Keywords:** overhead athletes, Kerlan-Jobe Orthopaedic Clinic Shoulder & Elbow Score, translation, cross-cultural adaptation, validity, reliability, European Portuguese version

## Abstract

**Background/Objectives**: The Kerlan-Jobe Orthopaedic Clinic Shoulder & Elbow Score (KJOC) is used to identify dysfunctions and estimate injury risk in overhead sports athletes. Although it has been validated in several countries, a European Portuguese version is currently unavailable. This study aimed to translate, culturally adapt and assess psychometric properties (validity and reliability) of the European Portuguese KJOC (KJOC-PT). **Methods**: The KJOC-PT was translated and culturally adapted according to international guidelines. One hundred athletes were selected (median age 24 [IQR 17] years, 72% male) and divided into two groups: asymptomatic and symptomatic athletes. The convergent validity was assessed by correlating the KJOC-PT with the Disabilities of the Arm, Shoulder and Hand (DASH) and DASH-Sports. 31 athletes from the initial sample were considered to assess between-day reliability and agreement (Bland-Altman analysis). Floor and ceiling effects were also calculated. Sampling adequacy was assessed using the Kaiser Meyer-Olkin (KMO) test. **Results**: Minor cultural and linguistic changes were made in the KJOC-PT. This version demonstrated excellent internal consistency (Cronbach’s α = 0.91 to 0.97) and moderate negative correlations for validity (KJOC-PT with DASH, rho = −0.595; with DASH-Sports, rho = −0.533, both *p* < 0.001). Good reliability (ICC_2,1_ = 0.77 to 0.89 [95%CI 0.36 to 0.96]), measurement error (SEM = 4.11 to 6.90; MDC = 11.39 to 19.13) and mean difference ranging from −0.08 ± 6.14 to 3 ± 9.17 were found. No floor effect (0%) and ceiling effects of 24.2% for the total sample (50% for asymptomatic and 5.1% for symptomatic athletes) were found. **Conclusions**: KJOC-PT is now available and is a valid and reliable instrument for use by athletes in overhead sports.

## 1. Introduction

Overhead sports include movements or sports activities performed with the upper limb raised above the head [1]. In this sports environment, there is a need to quantify, in a practical and rapid way, the degree of disability and risk associated with sports practice, as well as to estimate a cut-off to facilitate decision-making regarding the athlete’s performance. For this purpose, the Kerlan-Jobe Orthopaedic Clinic Shoulder & Elbow Score (KJOC) questionnaire [2] has been frequently applied to different overhead athletes with dysfunctions in the shoulder and elbow joint complex. The KJOC is a valuable instrument because it captures subtle performance limitations that general shoulder assessment tools often miss. While tools such as the Shoulder Pain and Disability Index (SPADI), Constant Score, and Disabilities of the Arm, Shoulder and Hand (DASH) were developed for the general population or specific clinical conditions, the KJOC provides a more detailed, sports-specific, and sensitive evaluation. This makes it better suited to detect performance-related dysfunctions in athletic populations [2].

This instrument has been used in various sports. Kraeutler et al. (2013), Painter et al. (2024) and Leenen et al. (2024) used it to identify the clinical history of upper limb injuries, describing musculoskeletal symptoms and athletes’ functionality, particularly in baseball players [3,4,5]. Wilkins et al. (2023) used the KJOC questionnaire score to identify possible differences in the risk of overuse injuries among young athletes with and without early sports specialization [6]. Michelin et al. (2022) applied the KJOC questionnaire in cases of elbow joint injuries post-arthroscopy, particularly in instances of capitellar microfracture in adolescent baseball players, gymnasts and cheerleaders, assessing the severity and location of the injury, range of motion and time to return to sports activity [7]. A literature review by Jackson et al. (2023) gathered data to estimate the return-to-sport ratios and complications after cubital collateral ligament repair in young athletes [8], as did Tsuruike et al. (2018), Steffes et al. (2022) and Desai et al. (2023) with professional baseball players [9,10,11]. The sample in Desai et al. (2023) [11] also included baseball players with elbow impingement who had undergone posteromedial arthroscopy for osteophyte resection.

Regarding shoulder joint injuries, Faherty et al. (2019) applied the KJOC questionnaire in order to determine the predictive capacity of musculoskeletal injury characteristics in the upper limb, considering function and pain, in baseball players [12]. Undoubtedly, the high velocity and range of motion overhead in baseball places a great deal of strain on the glenohumeral (GU) joint, often leading to pain and instability. Accordingly, Wardell et al. (2022) used the KJOC questionnaire to identify possible correlations between glenohumeral joint instability (capsular laxity), glenohumeral joint hypermobility (partial capsular laxity), normal glenohumeral joint (no capsular laxity), and the presence of pain exclusively during baseball throws [13]. Brzoska et al. (2023) analyzed professional athletes from different sports (both overhead and non- overhead, contact and non-contact), all with anterior shoulder instability, some of whom had undergone open surgery (Latarjet) and other arthroscopies [14]. They used the KJOC questionnaire to assess the sports activity level of the athletes who underwent each surgical procedure. Similarly, but with patients presenting posterior shoulder instability who underwent arthroscopic capsulolabral repair, Karpinski et al. (2023) used the questionnaire to compare the clinical outcomes between two different surgical techniques [15]. In a systematic review [16], Migliorini et al. (2023) analyzed the KJOC questionnaire in overhead athletes with rotator cuff injuries following arthroscopic repair to describe self-reported clinical changes, time and level of return to sports activity, as well as the complication rates.

Recent literature demonstrates that the KJOC score effectively discriminates between symptomatic and asymptomatic athletes across various overhead sports. The score correlates with subjective competitive level and sport-specific functional impairment [17]. Additionally, the KJOC score is closely linked to a history of upper extremity musculoskeletal injuries, as well as self-perceived pain and function. This underscores the score’s utility in clinical assessments and research studies involving collegiate and professional athletes [12].

Given the existence of various upper limb injuries in overhead sports athletes, and the need to assess, in a simple and accessible way, the functionality of athletes with injuries affecting the shoulder and elbow during sports practice, validated translations of the KJOC are available in several languages, namely Italian [18], Korean [19], Turkish [20], Norwegian [21], Persian [22], Dutch [23], German [24], Finnish [25], Ukrainian [26], Spanish [27], Japanese [28], Greek [29] and Brazilian Portuguese [30]. However, there is currently no European Portuguese version available.

Therefore, the aim of this study is to translate and culturally adapt the KJOC questionnaire into European Portuguese, as well as to analyze the psychometric properties (reliability and validity) of the European Portuguese version (KJOC-PT).

## 2. Materials and Methods

### 2.1. Study Design

The protocol of the present study was submitted to and approved by the Ethics Committee of the School of Health Fernando Pessoa (reference ESS/LFST-720/25, 11 March 2025). Authorization to carry out the process of translating and culturally adapting the KJOC in European Portuguese was requested from Frank Alberta, author of the original version [2] and was granted.

This cross-sectional study was conducted in 2 phases: the first consisted of the translation and cultural adaption of the KJOC questionnaire from English to European Portuguese, and a second phase, which involved the assessment of the internal consistency, convergent validity and reliability of the KJOC-PT applied to overhead athletes from different sports.

### 2.2. Translation and Cultural Adaptation

The methodology for translation and cultural adaptation followed the guideline of Beaton et al. (2000), involving 6 stages: (1) initial translation; (2) synthesis of the translation; (3) back-translation; (4) expert committee; (5) pre-test of the final version; and (6) approval by the expert committee [31]. To ensure both technical accuracy and clarity of everyday language, the initial translation from English to European Portuguese was performed by two bilingual native Portuguese translators—one with a background in health (physiotherapist) and one without (Portuguese teacher). An expert panel then synthesized their independent translations into one version. The panel consisted of researchers who had prior experience with translation, cultural adaptation, or assessing the psychometric properties of instruments.

Following, two native English translators (one a professional translator and the other an English teacher, both of whom are fluent in Portuguese) who were unfamiliar with the original questionnaire and who did not have health expertise performed the back-translation of the synthesized Portuguese version. This step aimed to verify that the Portuguese version accurately reflected the original’s concepts.

### 2.3. Participants

The sample was a non-probabilistic, convenience-based, using the “snowball” sampling technique, with the aim of maximizing the number of participants, given the difficulties inherent in identifying and accessing the target group. The researchers directly contacted athletes from overhead sports, who were considered eligible for the study. Subsequently, these athletes were asked to share the study with other practitioners of the same sport through their personal and digital contact networks, establishing recruitment chains that allowed for the expansion of the sample. Data was collected using an online questionnaire made available via a Google Forms link.

At the beginning of the study, all potential participants were duly informed and clarified about its objectives and possible future benefits. Those who agreed to participate were asked to provide informed consent in accordance with the Declaration of Helsinki. They were also asked to indicate their availability to complete the questionnaire again one week later.

Two groups of athletes were defined: group 1—asymptomatic athletes who practice their sport, without shoulder or elbow injuries/complaints; and group 2—symptomatic athletes who are still able to continue practicing their sport, despite shoulder or elbow injuries/complaints.

### 2.4. Eligibility Criteria

The inclusion criteria were as follows: participants had to be athletes from overhead sports, aged 18 or over, male or female, athletes with or without symptoms in the shoulder or elbow joint complex. Athletes with symptoms had to be experiencing symptoms at the time, with no indication of how long the symptoms had been present. The study also included athletes of all levels of competence and practice who understood the purpose of the study and had given their informed consent. The exclusion criteria were as follows: history of upper limb injury or any other injury that prevents the athlete from actively participating in their sport [25], occurrence of a new injury during the study’s reliability assessment interval [22], not being familiar with the European Portuguese language.

### 2.5. Instruments and Variables

Sociodemographic (age, sex, weight, height, body mass index [BMI]), sports practice (training hours, sports, level of practice, types of injuries) data, as well as training and injury-related characteristics, were collected using the online questionnaire specifically developed for this study.

The KJOC questionnaire was applied and aims to assess the functionality of the upper limb, more specifically the shoulder and elbow, in athletes who practice overhead sports. It consists of 10 questions (Q1–Q10), with a score ranging from 0–100 points. The closer the score is to 100, the higher the athlete’s functionality [2].

In addition, the Disabilities of the Arm Shoulder and Hand Outcome Measure (DASH) was used and consists of 30 items that make it possible to analyze the degree of difficulty in performing certain functions during the previous week. In addition, there are 4 optional items focused on the sports component [32,33]. Authorization to use the questionnaire was requested from the author of the European Portuguese version [33], which was granted.

### 2.6. Data Analysis

Data was analyzed using Statistical Package for Social Sciences (SPSS) software, version 29.0 for Windows. The significance level used was 0.05. The Shapiro-Wilk test was applied to assess the normality of the sample. Categorical variables were described using absolute and relative frequencies, and continuous variables were described using mean and standard deviation (SD) or median and interquartile range (IQR), according to the normality of the distribution. Inter-group comparison of quantitative variables was carried out using the independent samples *T*-Test or the Mann-Whitney test, depending on the normality of the sample data. The Wilcoxon test was used to compare the test and retest scores of the KJOC-PT.

The internal consistency of the KJOC-PT questionnaire was determined using the Cronbach’s alpha coefficient (α: >0.9, excellent; 0.8–0.9, good; 0.7–0.8, acceptable; 0.6–0.7, questionable; 0.5–0.6, poor; <0.5, unacceptable) [34,35]. The applicability of principal component analysis was assessed using the calculating index Kaiser-Meyer-Olkin-KMO (measure of sampling adequacy index). According to recommendations, this index should have values higher than 0.6 in order to apply principal component analysis [36]. The Bartlett test, which compares the correlation matrix with the identity matrix, was also used. A significant result (*p* < 0.05) indicates that the variables are correlated, demonstrating suitability for Principal Component Analysis.

Convergent validity was calculated using the Spearman correlation (rho) between the total scores of the KJOC-PT and the DASH, as well as between the KJOC-PT and the DASH-Sports, for the entire sample and for the subgroups (asymptomatic and symptomatic athletes). The strength of these correlations was also analyzed: significant correlation coefficients of 0–0.10 as unacceptable; 0.10–0.39 as weak; 0.40–0.69 as moderate; 0.70–0.89 as strong; 0.90–1.00 as very strong) [37].

Test-retest reliability was conducted with a time interval of 1 week and estimated using the ICC model 2 (random two-way effects), with a single rater (ICC_2,1_) with a 95% confidence interval (CI) for the total score and for each item in the KJOC-PT test and retest. ICC_2,1_ value below 0.50 indicates poor reliability, between 0.50–0.75 moderate reliability, between 0.75–0.90 good reliability, and values > 0.90 excellent reliability [38].

Measurement errors were obtained using the standard error measurement (SEM) and the minimal detectable change (MDC) with 95% CI. The SEM and %SEM were calculated using the following formulas:

SEM = SD × √(1 − ICC),(1)

where SD is the standard deviation obtained in the two response moments of the KJOC-PT.

%SEM = (SEM/mean) × 100,(2)

where “mean” is the average of the results obtained at the two moments. High SEM values indicate lower questionnaire accuracy [39]. MDC95 and %MDC95 were calculated using the following formulas:

MDC95 = 1.96 × √2 × SEM,(3)

%MDC95 = (MDC95/mean) × 100,(4)

where “mean” is the average of the results obtained at the two moments. %MDC < 30% is considered acceptable [40,41].

A Bland-Altman (B&A) plot with 95% CI limits of agreement was used to show the mean difference between the results obtained in the test and retest, in order to compare them with the mean of the two time points. In addition, the B&A limits of agreement (LoA) with 95% CI were calculated using the following formula:

LoA = mean_difference_ ± 1.96 × SD_difference_, (5)

where mean_difference_ and SD_difference_ are the mean and SD of the differences between the two moments [42,43].

The verification of the floor or ceiling effect was based on the percentage (≥15%) of participants who obtained the minimum or maximum scores for each item. Floor and ceiling effect were calculated using the following formulas [44]:

%Floor effect = n participants with minimum score/total n participants,(6)

%Ceiling effect = n participants with maximum score/total n participants,(7)

## 3. Results

### 3.1. Translation and Cultural Adaptation

The pre-test phase involved a total of 10 overhead athletes and 7 healthcare professionals to verify the adequacy of the questions and answers.

Given the specificity of overhead sports, it was necessary to adapt certain terms to the European Portuguese context so that the KJOC could be easily understood by athletes from all sports disciplines. Therefore, in the athlete characterization section, the word “speciality” (“*especialidade*”) was paired with “position”, as there are sports such as swimming, athletics and gymnastics that are subdivided by speciality rather than position. At the beginning of the questionnaire, when referring to the “arm”, the phrase “(shoulder and elbow) injury” (“*ombro e cotovelo lesionado*”) was added. The word “games” was supplemented with “competition” (“*competição*”) in order to encompass a wider variety of sports. Furthermore, regarding linguistic and cultural adaptations, adjustments were made to the item originally described using the North American league system “Professional Major League”, “Professional Minor League”, “Intercollegiate” and “High School”, which had to be adapted to the European Portuguese league system “*Desporto profissional*”, “*Semi-professional*”, “*Amador*”, “*Desporto Universitário*” and “*Desporto escolar no ensino secundário*”, respectively. The term “play” was replaced with “practice” (“*praticar*”), and the expression “get loose” in question Q1, was replaced with being “fit/prepared” (“*apto/preparado*”). Finally, in Q9, it was suggested to add the words “support” and “sporting gesture” translated from “*apoio*” and “*gesto desportivo*”, respectively, due to the specific movements used in certain overhead sports. All changes were requested and approved by the original author. After this authorization, the final European Portuguese version (KJOC-PT) was produced. KJOC-PT is available upon request.

### 3.2. Characterization of the Sample

A total of 102 overhead athletes were included in the study, but 2 were excluded because they did not complete the questionnaires. The median age of the participants was 24 [IQR 17] years. The eligible sample consisted of 100 athletes, subdivided into two groups: asymptomatic athletes (30%) and symptomatic athletes (70%) (Table 1). The sample presented homogeneous characteristics between athletes with and without pain symptoms (0.414 < *p* < 0.608), and was predominantly male, both in the total sample (72%) and when subdivided into the two groups (73.3% and 71.4%). A significant difference was found in the number of weekly training hours between athletes without pain and those with pain (*p* < 0.001), with athletes experiencing pain training for a greater number of hours per week. Athletes who reported pain had significantly lower KJOC-PT scores (*p* = 0.003) and higher DASH scores (*p* = 0.018) (Table 1).

The participants practiced a total of 12 different sports. The highest responses came from volleyball (20.6%), followed by handball (18.6%) and paddle tennis (17.6%). These sports also exhibited the highest frequency of pain symptoms (22.9%, 18.6%, and 12.9%, respectively). The proportion of amateur athletes was 45.1%, and this group reported the highest frequency of pain (41.4%). An analysis of the reported injuries revealed that shoulder tendinopathy was the most frequent complaint (28.4%) (Table A1).

### 3.3. Internal Consistency of the KJOC-PT

The KJOC-PT demonstrated excellent internal consistency (≥0.9) both in the total sample of 100 athletes and in the subgroups of asymptomatic and symptomatic athletes, with Cronbach’s alpha values of 0.94, 0.97 and 0.91, respectively. Among the 10 questions on the KJOC-PT, Q1 showed the lowest corrected item-total correlation (0.42) in the total sample. Excluding Q1 would increase the alpha value to 0.94 (Table A2).

### 3.4. Validation of the Internal Structure of the KJOC-PT

The validation of the internal structure was examined using principal component analysis of the 10 items. The KMO measure (KMO = 0.916) and the Bartlett test confirmed the adequacy of applying this questionnaire to the present sample (*p* value < 0.001).

### 3.5. Convergent Validity of the KJOC-PT

DASH scores (rho = −0.595, rho = −0.449 and rho = −0.612) and DASH Sports scores (rho = −0.553, rho = −0.602 and rho = −0.511) were significantly, negatively and moderately correlated with KJOC-PT scores in the total sample, asymptomatic athletes and symptomatic athletes, respectively (*p* < 0.001, except for the correlation between DASH and KJOC-PT in asymptomatic athletes, which had a *p* value of 0.013).

### 3.6. Reliability (Between-Day Test-Retest, Measurement Error and Agreement)

The reliability assessment included 31 athletes (64.5% male, 61.3% symptomatic and 38.7% asymptomatic). No significant differences were observed between the two moments when comparing the total mean scores of KJOC-PT (*p* = 0.348) and the scores from each question (Q1–Q10) from the test and retest, both for the total sample and for the two subgroups (symptomatic and asymptomatic athletes) (0.071 < *p* < 0.725).

The KJOC-PT total score presented good reliability (total sample: ICC_2,1_ = 0.83 [0.77–0.94] and Cronbach’s α = 0.94; asymptomatic athletes: ICC_2,1_ = 0.77 [0.36–0.93] and Cronbach’s α = 0.86 and symptomatic athletes: ICC_2,1_ = 0.89 [0.74–0.96] and Cronbach’s α = 0.94). The ICC_2,1_ values and Cronbach’s α for each question (Q1–Q10) of the KJOC-PT for the total sample and for symptomatic and asymptomatic athletes are presented in Table A3.

A SEM value of 6.90 (%SEM = 7.9%) and an MDC95 value of 19.13 (%MDC95 = 21.8%) were obtained for the total sample. In the group of asymptomatic athletes, the SEM value was 4.11 (%SEM = 4.38%) and the MDC95 value was 11.39 (%MDC95 = 12.13%). In the group of symptomatic athletes, the SEM value was 6.56 (%SEM = 7.83%) and the MDC95 was 18.18 (%MDC95 = 21.7%).

**Figure 1:**
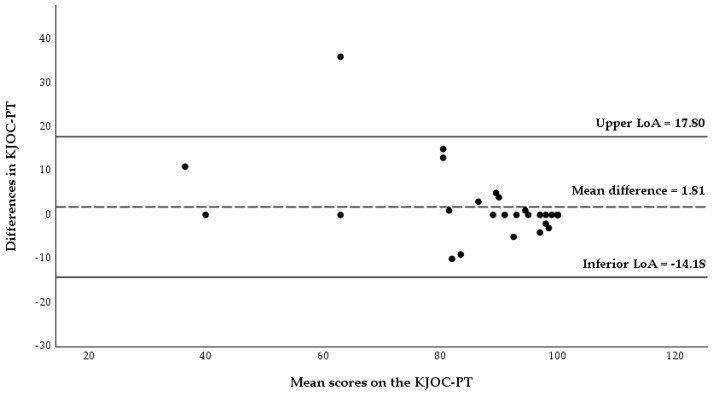
Bland-Altman plot of the difference between KJOC-PT scores against the mean KJOC-PT scores in total samples (n = 31). Abbreviations: LoA, limits of agreement.

**Figure 2:**
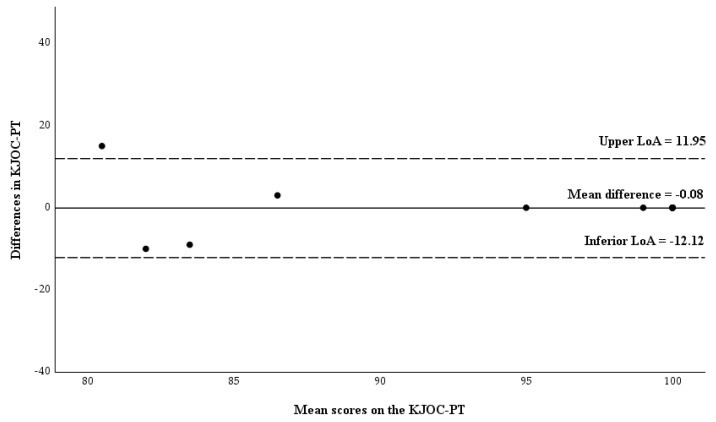
Bland-Altman plot of the difference between KJOC-PT scores against the mean KJOC-PT scores in asymptomatic athletes (n = 12). Abbreviations: LoA, limits of agreement.

**Figure 3:**
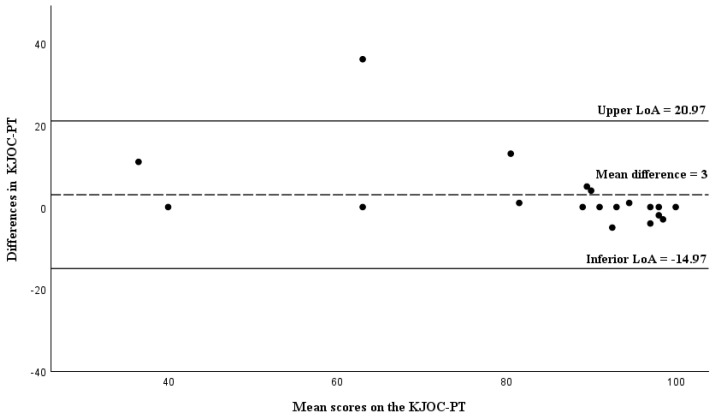
Bland-Altman plot of the difference between KJOC-PT scores against the mean KJOC-PT scores in symptomatic athletes (n = 19). Abbreviations: LoA, limits of agreement.

The LoA plots showing the differences between the first and second KJOC-PT scores vs. the mean of the scores of the first and second KJOC-PT for the total sample, and for each subgroup are shown in Figure 1, Figure 2 and Figure 3. For the total sample, the mean bias (i.e., the average of the differences, mean_diff_) was 1.81 with LoA ranging from −14.18 to 17.8. Measurements from one participant fell outside the upper limit of the LoA. No evidence of systematic error was found (Figure 1).

For the asymptomatic athletes, the mean bias was −0.08 with LoA ranging from −12.12 to 11.95. Measurements from one participant fell outside the upper limit of the LoA. No evidence of systematic error was found (Figure 2).

For the symptomatic athletes, the mean bias was 3 with LoA ranging from −14.97 to 20.97. Measurements from one participant fell outside the upper limit of the LoA. No evidence of systematic error was found (Figure 3).

### 3.7. Floor and Ceiling Effect

No floor effect was observed in either the total sample or the asymptomatic and symptomatic athletes, as none of the participants obtained the minimum score. Regarding the ceiling effect, it was found that 24.2% (n = 7) of the total sample achieved the maximum score, 50% (n = 6) among asymptomatic athletes and 5.1% (n = 1) among symptomatic athletes.

## 4. Discussion

In this study, the KJOC questionnaire was translated and culturally adapted into European Portuguese, and it demonstrated to be a valid and reliable measure for assessing shoulder and elbow function and performance in Portuguese athletes.

### 4.1. Translation and Cultural Adaptation

The process of translating and culturally adapting the original English version of the KJOC into European Portuguese proceeded without major difficulties. However, some linguistic and cultural adjustments were required. The structure of the KJOC was preserved, with all the items incorporated into the European Portuguese version, designated as KJOC-PT, covering essential areas for athletes who practice overhead sports in European Portuguese-speaking contexts. During the translation into European Portuguese and, subsequently, in the back-translations into English, minor linguistic inconsistencies were identified, which were resolved at a meeting with the expert committee. Nonetheless, authorization was requested from the original author, Frank Alberta, to adapt certain concepts to the European Portuguese reality, including both linguistic adaptations and sport-specific particularities inherent to each overhead sport.

### 4.2. Internal Consistency

The results obtained indicate that the European Portuguese version (KJOC-PT) presents excellent internal consistency in the study population (Cronbach’s α = 0.94). Noteworthy that this value is similar to those reported in other versions, which ranged from 0.84 [23] to 0.98 [26].

Question (Q) 1 presented a lower corrected item-total correlation compared with the others (0.422), which may be reducing the reliability of the questionnaire. By excluding Q1, the alpha value would increase (0.94), while maintaining the same classification (Table A2). Therefore, Q1 was not excluded from the present study, consistent with several previously translated versions [19,20,22,28]. This finding can be explained by the variability in the participants’ response patterns to Q1, most likely due to the difficulty in understanding the content of the question (How difficult is it for you to get loose or warm prior to competition or practice?). The expressions “*apto ou aquecido*” (fit or warmed up) or “being prepared” for competition are two subjective concepts, influenced by multiple factors, physical, mental and environmental, rather than solely by the presence of shoulder and/or elbow injury. This question raised the greatest interpretation difficulties during the content validity process, which involved 7 health professionals and 10 athletes. Despite the adaptation of the expression “to get loose or warm” into “*estar apto ou aquecido*”, considering Portuguese sports culture and habits, this change did not achieve the desired effectiveness.

It is noteworthy that our internal consistency values are similar to those reported in other validated versions. However, it was slightly lower than the values reported for the Norwegian (0.95) [21], Greek (0.95) [29] and Ukrainian (0.96–0.98) [26] versions. It was comparable to the Turkish version (0.94) [20] and the German version (0.93) [24], and slightly higher than the Dutch version (0.84) [23], the Finnish version (0.92) [25], the Persian version (0.92) [22], the Italian version (0.91) [18] and the Japanese version (0.91) [28].

### 4.3. Convergent Validity

In this study, significant, moderate and negative correlations were observed between the different questionnaires, not only when considering the entire sample, but also when analyzing symptomatic and asymptomatic athletes. The fact that the correlation between the KJOC-PT and the DASH was moderate in the group of asymptomatic athletes, can be explained by the fact that these two questionnaires focus more on pain symptoms and on the partial or total inability to perform sports sporting gestures. In other words, they are more targeted toward athletes with upper limb complaints, namely involving the shoulder and elbow, and preferably those engaged in competitive sports. When compared with previous studies, the KJOC-PT shows a correlation between the KJOC and DASH (rho = −0.595) and DASH-Sports (rho = −0.553), values that fall within the ranges reported in previous versions: DASH: r = [−0.757; −0.43] [25,29], and DASH-Sports: r = [−0.84; −0.40] [20,29]. However, it demonstrates a lower correlation compared to the original version, namely r = 0.81 with DASH and r = 0.85 with DASH-Sports [2].

Compared with previous validated versions of the KJOC, the KJOC-PT demonstrated slightly lower correlations than the Italian (DASH: r = −0.697; DASH-Sports: r = −0.704) [18], Turkish (DASH: r = −0.64; DASH-Sports: r = −0.84) [20], Norwegian (DASH: r = −0.642; DASH-Sports: r = −0.790) [21], and Finnish (DASH: r = −0.757; DASH-Sports: r = −0.667) [25] versions. On the other hand, the KJOC-PT demonstrated higher correlations than the German (DASH: r = −0.50; DASH-Sports: r = −0.54) [24], Persian (DASH-Sports: r = −0.559) [22], Japanese (DASH: r = −0.581) [28], and Greek (DASH: r = −0.43; DASH-Sports: r = −0.40) [29].

### 4.4. Reliability: Test-Retest

According to Terwee et al. (2007), a 1-week interval between test and retest was. This period should be long enough to prevent the respondent from remembering their previous answers, but short enough to avoid clinical changes [35]. The test-retest was conducted with 31 of the 100 participants and revealed good reliability, not only for each individual item, but also for the final score. When analyzing the subdivided sample good, reliability was observed in both asymptomatic and symptomatic athletes (ICC_2,1_ = 0.77 and ICC_2,1_ = 0.89, respectively). Thus, the results fall within the ICC_2,1_ range of previously translated versions, ICC = [0.77–0.99], with the lowest ICC being that of the Finnish version [25] and the highest that of the Italian version [18].

However, Q1 of the KJOC-PT proved to be an exception, showing poor reliability (ICC_2,1_ = 0.549 in the total sample and ICC_2,1_ = 0.492 in symptomatic athletes). This finding is consistent with earlier observation regarding the low comprehensibility of this question. Poor reliability for this question is also reported in some other versions, such as the Finnish (ICC = 0.380) [25] and the Japanese (ICC = 0.493) [28].

Among asymptomatic athletes, some questions showed poor ICC_2,1_ values (Q4) or moderate reliability (Q2, Q6 and Q9). All these questions are related to situations of complaints/injuries (Q2 and Q4) and change/control of movements/sports activity (Q6 and Q9). Since asymptomatic athletes did not present complaints at the time of response, it was more difficult for them to recall the limitations experienced during previous shoulder and/or elbow injuries (injury history), which may have led to less accurate results.

When compared with previous versions, our results are consistent with the original questionnaire (English version), which presented an ICC = 0.88 [2], as well as with the Italian (ICC = 0.99) [18], Norwegian (ICC = 0.97) [21], Spanish (ICC = 0.96) [27], Greek (ICC = 0.95) [29], German (ICC = 0.94) [24], Korean (ICC = 0.94) [19], Turkish (ICC = 0.93) [20], Japanese (ICC = 0.87) [28], Persian (ICC = 0.82) [22] and Finnish (ICC = 0.77) [25] versions.

### 4.5. Measurement Error

The SEM and MDC95 provide a more detailed description of measurement errors, offering important data regarding the accuracy and reliability of the KJOC-PT. A SEM of 6.90 (%SEM = 7.9%) was obtained, which corresponds to a relatively low value, thus indicating good representativeness of the study sample in relation to the average population of overhead athletes with similar characteristics. In the future, the questionnaire may therefore be used with reasonable confidence, allowing inferences to be made about the target population.

### 4.6. Agreement: Bland-Altman Analysis

The Bland-Altman method is a tool for assessing agreement, and the fact that the mean difference between test and retest was low (1.81) indicates little bias with no evidence of systematic error. However, among symptomatic athletes, responses showed greater bias compared to the responses of asymptomatic athletes (3 vs. −0.08). Values closer to 0 suggest stronger agreement [42]. Nevertheless, nearly all the respondents fell within the limits of agreement. When compared to the other versions, the mean value of the test-retest differences for the total KJOC-PT sample was higher than that reported in the German version (−0.4) [24], the Finnish version (−0.22) [25] and the Japanese version (−1.8) [28].

### 4.7. Floor and Ceiling Effect

No floor effect was found in the KJOC-PT, nor in any of the previously translated versions [18,20,23,24,25,29]. However, the ceiling effect was observed in the KJOC-PT, with 24.2% of the total sample reaching the maximum score. This high value may be related to the combined analysis of the two subgroups, since, when analyzing the ceiling effect in asymptomatic athletes, the value was markedly higher (50%), meaning that this group frequently scored the maximum (10 points) on each item. The high ceiling effect among asymptomatic athletes suggests that a large percentage of high-functioning athletes earn the maximum score. This limits the instrument’s ability to detect subtle improvements or declines in upper extremity function within this subgroup. This directly impacts on the responsiveness of the KJOC-PT, as further gains in function or minor decrements cannot be captured when scores cluster at the upper limit. Consequently, the KJOC-PT may be less useful for monitoring or assessing outcomes in elite, asymptomatic athletes, for whom subtle changes in performance are clinically relevant.

In contrast, no ceiling effect was observed among symptomatic athletes (5.1%). This difference in floor and ceiling effects between asymptomatic and symptomatic athletes has also been reported in some previously translated versions [20,23,24,25]. If the ceiling effect (≥15%) was present in symptomatic athletes, this would suggest that the participants had reached the maximum score on several items of the KJOC. In such a situation, it would be pertinent to reconsider the questions in order to ensure greater specificity in capturing the impairments caused by injuries, both at a structural and sporting level. In other words, all complaints from athletes should be more rigorously assessed, as well as any interruptions or modification in sporting activity. Another issue of the presence of the ceiling effect in symptomatic athletes would be its impact on evaluating improvements, since these athletes would already be scoring at the maximum level on the questionnaire items.

### 4.8. Limitations of the Study

One limitation of this study is that the questionnaire is self-reported, which introduces several potential sources of bias. These include perception, memory, and reporting biases, which are influenced by athletes’ motivation, mood, and desire to present themselves in a socially acceptable manner.

The digital adaptation of the instrument, administered via Google Forms, may also contribute to measurement bias. Because the platform does not allow respondents to place an “X” on a continuous line as in the original version [2], a 10-point numerical scale was used, which may reduce the sensitivity of responses [19,28]. Furthermore, completing the questionnaire online may introduce additional biases such as variations in device type (e.g., phone vs. computer), screen size and environmental distractions while responding. Differences in digital literacy among participants could also influence how items are interpreted or answered. Collectively, these factors may affect the accuracy and consistency of the reported data.

Regarding reliability assessment (test-retest), the sample size was small and lower than that recommended in the literature [35], which may have affected the results obtained. Another possible limitation in this analysis is the athletes’ tendency to adjust their answers between the 2 moments. Additionally, the responsiveness of the KJOC-PT to a specific injury or to a specific overhead sport was not investigated.

### 4.9. Implications for Clinical and Sport Contexts and Suggestions for Future Research

The KJOC has important implications in both clinical and sports settings. It can serve as an early indicator of injury risk and help predict an athlete’s readiness to return to sports. When an athlete scores below 90, clinicians and sports trainers should treat this as a warning sign that the athlete may be performing with pain, an underlying injury, or reduced capacity [3]. Therefore, routinely monitoring KJOC scores in European Portuguese contexts can guide training adjustments, inform rehabilitation decisions, and help safeguard athlete health and performance.

Future studies should assess the longitudinal responsiveness of the KJOC-PT by examining its ability to detect expected changes in the construct over time in overhead athletes after an intervention. This should be done using an appropriate design with predefined hypotheses about the direction and magnitude of change [45]. It is also recommended that studies apply the KJOC-PT to specific sports, thereby enhancing the adaptation and interpretation of the questionnaire. This would increase the contribution of this simple, cost-effective and accessible assessment tool, enabling healthcare professionals and coaches to better understand athletes’ clinical conditions and to develop preventive strategies.

## 5. Conclusions

This study demonstrates that the European Portuguese version of the KJOC is now available and valid and reliable for assessing shoulder and elbow function in Portuguese people engaged in overhead sports.

## Figures and Tables

**Table 1:** Characteristics of participants.

	Total of Athletes (n = 100)	Asymptomatic Athletes (n = 30)	Symptomatic Athletes (n = 70)	*p* Value
Age (years)	24 [17]	23.5 [13]	24 [20]	0.592 *
Sex, male, n (%)	72 (72%)	22 (73.3%)	50 (71.4%)	0.122 ^#^
Body mass (kg)	77.3 ± 1.6	75.8 ± 3.1	77.9 ± 1.8	0.489 ^†^
Height (m)	1.8 ± 0.01	1.8 ± 0.02	1.8 ± 0.01	0.608 ^†^
BMI (kg/m^2^)	24.4 [4.1]	23.7 [4.6]	24.5 [4.2]	0.414 *
Training hours (hours/week)	7 [7.4]	5 [2.3]	8 [7]	<0.001 *
KJOC-PT (score)	92 [16]	98 [12]	92 [17]	0.003 *
DASH (score)	1.7 [5.8]	0 [2.7]	1.7 [7.5]	0.018 *
DASH Sports (score)	0 [5–12]	0 [3.1]	0 [14.1]	0.057 *

The values are expressed as mean ± standard deviation and median [interquartile range], unless otherwise indicated. * Mann-Whitney test, # Chi-square test, † independent samples T-test. Abbreviations: BMI—Body Mass Index; KJOC-PT—Kerlan–Jobe Orthopaedic Clinic Shoulder and Elbow Score (European Portuguese version); DASH—Disabilities of the Arm, Shoulder and Hand questionnaire.

## Data Availability

The data is available upon request from the corresponding author.

## Appendix A

**Table A1:** Sports, level of practice and types of injuries of the participants.

		Total Number of Athletes (n = 100)	Asymptomatic Athletes (n = 30)	Symptomatic Athletes (n = 70)
Sports	Handball	19 (18.6%)	6 (20.0%)	13 (18.6%)
	Basketball	7 (6.9%)	4 (13.3%)	3 (4.3%)
	Swimming	11(10.8%)	3 (10%)	8 (11.4%)
	Football	2 (2.0%)	0 (0.0%)	2 (2.9%)
	Volleyball	21 (20.6%)	5 (16.7%)	16 (22.9%)
	Futsal	1 (1.0%)	1 (3.3%)	0 (0.0%)
	Padel	18(17.6%)	9 (30.0%)	9 (12.9%)
	Tennis	1 (1.0%)	0 (0.0%)	1 (1.4%)
	Gymnastics	6 (5.9%)	0 (0.0%)	6 (8.6%)
	Rugby	1 (1.0%)	0 (0.0%)	1 (1.4%)
	Crossfit	9 (8.8%)	1 (3.3%)	8 (11.4%)
	Table tennis	4 (3.9%)	1(3.3%)	3 (4.3%)
Level of practice	Professional	19 (18.6%)	4 (13.3%)	15 (21.4%)
	Semi-professional	30 (29.4%)	8 (26.7%)	22 (31.4%)
	Amateur	46 (45.1%)	17 (56.7%)	29 (41.4%)
	University sports	2 (2.0%)	0 (0.0%)	2 (2.9%)
	School sports	3 (2.9%)	1 (3.3%)	2 (2.9%)
Types of injuries	Shoulder fracture	1 (1.0%)	-	1 (1.4%)
	Elbow fracture	0 (0.0%)	-	0 (0.0%)
	Dislocation	8 (7.8%)	-	8 (11.4%)
	Biceps brachii tendon rupture	0 (0.0%)	-	0 (0.0%)
	Rotator cuff tendon rupture	1 (1.0%)	-	1 (1.4%)
	Bone edema	1 (1.0%)	-	1 (1.4%)
	SLAP	2 (2.0%)	-	2 (2.9%)
	Bennett lesion	0 (0.0%)	-	0 (0.0%)
	Shoulder tendinopathy	29 (28.4%)	-	29 (41.4%)
	Epicondylitis	6 (5.9%)	-	6 (8.6%)
	Epitrochleitis	4 (3.9%)	-	4 (5.7%)
	Presence of intra-articular bodies	1 (1.0%)	-	1 (1.4%)
	Other injury	17 (16.7%)	-	17 (24.3%)

Abbreviations: SLAP—Superior labral anterior-posterior lesion.

**Table A2:** Internal Consistency of the KJOC-PT (n = 100).

KJOC	Corrected Total Item Correlation	Cronbach’s Alpha If Item Is Excluded
Q1	0.422	0.941
Q2	0.703	0.933
Q3	0.839	0.923
Q4	0.644	0.933
Q5	0.680	0.932
Q6	0.847	0.923
Q7	0.815	0.924
Q8	0.835	0.924
Q9	0.863	0.923
Q10	0.841	0.924

Abbreviations: Q—question.

**Table A3:** ICC values and Cronbach’s alpha of the KJOC-PT scores for each question.

KJOC-PT	Total Number of Athletes (n = 31)	Asymptomatic Athletes (n = 12)	Symptomatic Athletes (n = 19)
	ICC_2,1_ [95% CI] Cronbach’s α		
Q1	0.55 [0.24–0.76] 0.70	0.83 [0.52–0.95] 0.91	0.49 [0.46–0.77] 0.65
Q2	0.80 [0.62–0.90] 0.90	0.72 [0.31–0.91] 0.85	0.81 [0.58–0.92] 0.90
Q3	0.91 [0.83–0.96] 0.96	1 [-] 1	0.90 [0.77–0.96] 0.95
Q4	0.82 [0.66–0.91] 0.90	0.28 [−0.37–0.73] 0.42	0.89 [0.73–0.95] 0.94
Q5	0.80 [0.63–0.90] 0.89	0.08 [−0.57–0.63] 0.14	0.84 [0.63–0.93] 0.91
Q6	0.72 [0.50–0.86] 0.84	0.52 [−0.40–0.83] 0.68	0.70 [0.38–0.87] 0.83
Q7	0.91 [0.82–0.95] 0.95	0.65 [0.16–0.88] 0.78	0.92 [0.77–0.97] 0.97
Q8	0.92 [0.84–0.96] 0.96	0.90 [0.68–0.97] 0.94	0.92 [0.80–0.97] 0.96
Q9	0.84 [0.69–0.92] 0.91	0.63 [0.10–0.88] 0.76	0.85 [0.66–0.94] 0.92
Q10	0.95 [0.89–0.97] 0.97	0.89 [0.66–0.97] 0.94	0.95 [0.87–0.98] 0.97

Abbreviations: ICC—Intraclass Correlation Coefficient; Q—question.
